# Lung function in asbestos-exposed workers, a systematic review and meta-analysis

**DOI:** 10.1186/1745-6673-6-21

**Published:** 2011-07-26

**Authors:** Dennis Wilken, Marcial Velasco Garrido, Ulf Manuwald, Xaver Baur

**Affiliations:** 1 Institute for Occupational and Maritime Medicine, University Medical Center Hamburg-Eppendorf, Hamburg, Germany

**Keywords:** Asbestos, lung function, chest X-ray, computed tomography, meta-analysis

## Abstract

**Background:**

A continuing controversy exists about whether, asbestos exposure is associated with significant lung function impairments when major radiological abnormalities are lacking. We conducted a systematic review and meta-analysis in order to assess whether asbestos exposure is related to impairment of lung function parameters independently of the radiological findings.

**Methods:**

MEDLINE was searched from its inception up to April 2010. We included studies that assessed lung function parameters in asbestos exposed workers and stratified subjects according to radiological findings. Estimates of VC, FEV_1 _and FEV1/VC with their dispersion measures were extracted and pooled.

**Results:**

Our meta-analysis with data from 9,921 workers exposed to asbestos demonstrates a statistically significant reduction in VC, FEV_1 _and FEV_1_/VC, even in those workers without radiological changes. Less severe lung function impairments are detected if the diagnoses are based on (high resolution) computed tomography rather than the less sensitive X-ray images. The degree of lung function impairment was partly related to the proportion of smokers included in the studies.

**Conclusions:**

Asbestos exposure is related to restrictive and obstructive lung function impairment. Even in the absence of radiological evidence of parenchymal or pleural diseases there is a trend for functional impairment.

## Introduction

Asbestos fibres are one of the most pervasive environmental hazards because of their worldwide use in the last 100 years as a cheap and effective thermal, sound and electrical insulation material, especially in the construction, shipping and textile industries. The general public is also exposed to asbestos, mainly from deterioration and reconstruction or destruction of asbestos contaminated buildings, worn vehicle brake linings and from the deterioration of asbestos-containing products. In spite of outright bans or restrictions in nearly all industrialised countries nowadays, approximately 125 million workers are occupationally exposed to asbestos worldwide [1] and it is estimated that at least 100,000 die annually from complications of asbestos exposure [2]. In addition to mesothelioma, lung and laryngeal cancer, asbestos has long been known to cause non-malignant pleural fibrosis, (i.e. circumscript pleural plaques (PP), or diffuse pleural thickening (DPT)), pleural effusions, rounded atelectasis and lung fibrosis (asbestosis). Since inhalation of high doses of asbestos fibres may lead to a variety of functional impairments, the monitoring of workers who have been exposed to asbestos, particularly of their lung function, has gained in importance over the years. The identification of functional abnormalities is also relevant for compensation issues. While compromised lung function in pronounced disease is widely accepted, controversies still remain about a possible relationship between earlier or milder non-malignant asbestos-induced pleural or parenchymal fibrosis and reduced lung function measurements [3-11]. The American Thoracic Society and the American College of Chest Physicians [12,13], in particular, have lamented the lack of definitive knowledge in the prevalence and clinical relevance of asbestos-induced obstructive airway diseases and have determined to make this a priority for investigation and elucidation.

We have conducted a systematic review and a meta-analysis of the literature with the aim of identifying and quantifying alterations of lung function parameters in subjects occupationally exposed to asbestos. The leading question was whether occupational exposure to asbestos leads to impairments of lung function independently from the non-malignant radiological findings (i.e. normal chest radiograph (X-ray) or (high resolution) computed tomography (HR)CT, pleural plaques and diffuse pleural thickening or asbestosis).

## Materials and methods

### Selection criteria

We included publications that assessed lung function parameters and radiological imaging (chest X-Ray or (HR)CT) in persons with occupational exposure to asbestos. Only studies that applied an internationally accepted quality standard for lung function testing (i.e. ATS standard, ERS standard) and that provided information about the corresponding reference values or used reference group were considered. We included only studies reporting lung function parameters expressed as percent-predicted with a corresponding dispersion measure (i.e. standard deviation, standard error or confidence interval) and assigned them to one of the following radiological categories:

A. "Normal imaging", i.e. absence of pleural or lung parenchymal abnormalities.

B. "Pleural fibrosis", i.e. presence of pleural plaques and/or diffuse pleural thickening.

C. "Asbestosis", i.e. parenchymal fibrosis with or without pleural fibrosis.

To be included, studies had to provide data on the proportion of smokers among participants or on the dose (pack-years).

In a few potentially relevant studies the authors failed to report all information listed above (e.g. reference values, quality standards, dispersion measures), thus we tried to contact the authors in order to collect the missing data. Only three authors sent additional information that enabled us to include their publication in the meta-analysis.

### Search strategy

MEDLINE was searched from its inception to April 2010 via PubMed with the following search strategy:

("Asbestosis"[Mesh] OR ("Pleural Diseases"[Mesh] AND "Asbestos"[Mesh]) OR ("occupational exposure"[Mesh] AND "Asbestos"[Mesh]) OR ("Lung diseases"[Mesh] AND "Asbestos"[Mesh])) AND "Respiratory Function Tests"[Mesh] AND ("occupational diseases"[Mesh] OR "occupational health"[Mesh] OR "occupational exposure"[Mesh])

We applied the following PubMed limits in order to increase the specificity of our search:

("humans"[MeSH Terms] AND (English[lang] OR German[lang]) AND "adult"[MeSH Terms]) NOT ("Bronchoalveolar Lavage"[MeSH] OR "Neoplasms"[Mesh] OR "Case Reports "[Publication Type]).

Additionally, we scanned congress proceedings, reference lists of relevant articles and searched our own archive for further potentially relevant publications not identified through the electronic search.

### Data extraction

We extracted information on sample size, exposure to asbestos, proportion of non-smokers, radiological imaging method and lung function reference values together with the estimates for vital capacity (VC), forced expiratory volume in the first second (FEV_1_) and FEV_1_/VC with their corresponding SD, SE or 95% CI. Most of the studies reported forced vital capacity (FVC), but in some papers it was not clear whether FVC or slow (relaxed) vital capacity (SVC) was measured. Data were extracted by at least two of the authors independently from each other and discrepancies were solved by consensus after discussion. (HR)CT-based diagnoses were favoured over those based on X-rays when both were available.

### Data synthesis and statistical methods

We performed a meta-analysis to produce pooled estimates of VC, FEV1 and FEV1/VC for each of our designated radiological categories (A, B or C). Within each radiological category, we conducted subgroup analysis according to the type of imaging method used for the diagnosis (X-ray or (HR)CT).

Some studies reported results for different degrees of radiological impairments within the same category (e.g. different ILO scores for asbestosis). In these cases, we pooled the subgroup estimates from the same study with a fixed effects model to obtain a single estimate for each study within each radiological category (A-C).

A random effects model was used to calculate overall estimates for each radiological category.

We calculated I^2 ^as an indicator for the degree of heterogeneity across studies. Values of I^2 ^under 25% indicate low, up to 60% medium and over 75% considerable heterogeneity, making it advisable to perform the analysis using the random effects model [14]. In order to assess whether any observed between-study heterogeneity could be explained through study characteristics other than radiological imaging procedure, we also performed subgroup analysis for the proportion of never-smokers. For this purpose, we divided the study pool into two categories: studies with <25% of participants reporting to have never-smoked and studies with >= 25% of participants reporting to have never-smoked.

A second subgroup analysis was done for mean duration of asbestos exposure, dividing the study pool into two categories: studies reporting mean exposure duration longer than the median duration of the whole sample vs. studies with mean exposure duration shorter than median duration. In addition, we performed meta-regression analysis with the proportion of never-smokers and with the years of asbestos-exposed occupation.

All calculations were performed with the software Comprehensive Meta-Analysis 2.0. (Biostat™, Englewood, USA). Forest plot graphics were produced with Meta-Analyst Software [15]

## Results

**Figure 1:**
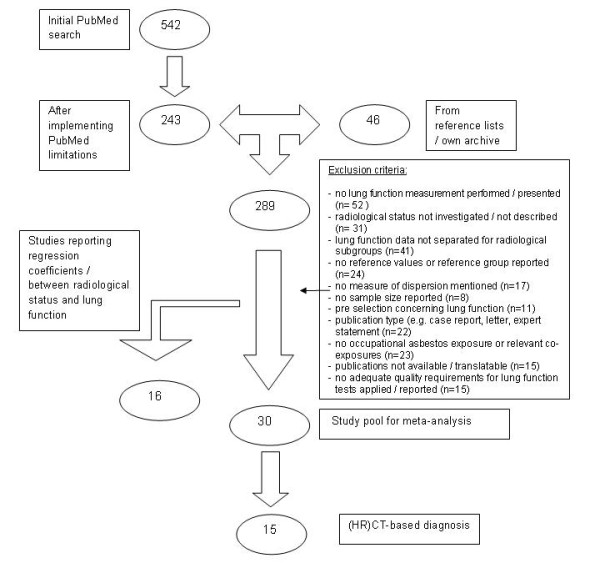
**Flow chart - Study selection process**.

A total of 542 papers were identified by the electronic literature database search and a further 46 papers through manual searching in congress reports, reference scanning and from our own archive (Figure 1). After scanning titles and abstracts, 289 articles were selected for a detailed assessment of the full publication. From these 289 articles, 30 met the inclusion criteria for the meta-analysis. The most frequent reasons for exclusion were lack of information about lung function parameters and/or about radiological diagnoses and lack of reporting statistical dispersion measures.

We included 27 cross-sectional studies, one case-control and two follow-up studies, comprising a total of 15,097 subjects of which the data for 9,921 were reported appropriately for inclusion in our meta-analysis. The characteristics of the included studies are shown in Table 1. Sample size ranged from 19 to 3,383. Some studies focussed on a specific occupation (e.g. asbestos manufacturing, insulation and cladding work, shipyard, asbestos industries, asbestos cement factory, ceiling tiles and wallboards, railway, ironworker, sheet metal, construction carpenters and millwrights) while others included subjects from different occupational fields. The mean duration of occupational exposure to asbestos was reported in 22 studies (i.e. 73% of the study sample) and ranged from 8.4 ± 6.1 to 32.7 ± 6.7 years (mean ± SD). The latency time (i.e. the time since first exposure) was reported in only 9 studies (i.e. 30%) and ranged from 24.5 ± 5.7 to 43.3 ± 6.7 years (mean ± SD). Estimations of asbestos fibre concentration (i.e. fibre-years) were reported only rarely [16,17].

**Table 1:** Characteristics of included studies

Reference	Study type	Study size	N (in meta-analysis)	Asbestos exposure					Smoking habits			Radiological chest imaging	Lung function	
						
				Occupation	Duration (yr)		Latency (yr)		non smokers (%)	Pack-years			Quality requirements	Reference values
					mean	SD	Mean	SD		mean	SD			
Ameille et al. 2004 [70]	CS	287	228	asbestos industry	25.8	9.4	33.2	9.4	38.1	nr	nr	HRCT	ATS 1987	ATS 1987
Begin et al. 1993 [71]	CS	61	46	asbestos industry	22.0	15.6§	nr	nr	21.3	28.0	23.4§	X-ray/HRCT	Bates 1971	Bates 1971
Begin et al. 1995 [72]	CS	207	96	diverse	26.0	13.7§	nr	nr	13.5	29.4	20.6§	X-ray/HRCT	Bates 1971	Bates 1971
Van Cleemput et al. 2001 [16]	CS	94	73	asbestos industry	25.0	1.4	nr	nr	15.0	10.9	20.6	HRCT	ECSC/ERS	Quanjer 1993
Delpierre et al. 2002 [55]	CS	97	38	asbestos industry	19.0	2.0	nr	nr	37.0	nr	nr	X-ray	Quanjer 1983	Quanjer 1993
Garcia-Closas and Christiani 1995 [60]	CS	631	541	construction/millwright	20.0	10.2	nr	nr	33.1	24.1	21.3	X-ray	ATS 1987	Crapo 1981
Hall and Cissik 1982 [24]	CS	135	113	diverse	#18.0	11.2	nr	nr	40.7	#21.2	19.5	X-ray	(ATS) OSHA 1978	Knudson 1983
Harkin et al. 1996 [73]	CS	107	37	diverse	nr	nr	32.5	9.5§	21.6	29.2	23.3§	X-Ray/HRCT	ATS 1986	Knudson 1983
Jarad et al. 1992 [74]	CS	60	60	diverse	10m	1-35r	34m	21-60r	13.3	21m	0-76r	X-Ray/HRCT	ATS 1979 (Cotes)	Cotes 1979
Kee et al. 1996 [75]	CC	1150	93	shipyard/construction	25.5	12.1	41	11.3	nr	23.9	25.7	HRCT	ATS 1987	Crapo 1981; ATS 1987
Kouris et al. 1991 [76]	CS	996	913	ceiling and wall	8.4	6.1	26.8	5.1	nr	17.6	19.1	X-ray	ATS 1979	Crapo 1981
Lilis et al. 1991 [59]*	CS	2790	1536	asbestos insulation	nr	nr	35.1	7.2§	46.6	nr	nr	X-ray	ATS 1987	ATS 1987
Nakadate et al. 1995 [77]	FU	242	27	asbestos industry	nr	nr	nr	nr	26.9	nr	nr	X-ray	ATS 1978	Pneumoconiosis law of Japan 1978
Neri et al. 1996 [25]	CS	119	38	diverse	10.9	6.1	24.5	5.7	26.3	14.0	11.9	X-Ray/HRCT	ATS 1987	Paoletti 1985
Niebecker at al. 1995 [9]	CS	382	194	diverse	nr	nr	nr	nr	28.9	nr	nr	X-ray	according to ERS/ATS	EGKS 1971
Ohar et al. 2004 [4]	CS	3383	3240	diverse	nr	nr	41.1	10.3	21.8	38.9	29.4	X-ray	ATS 1987	ATS 1987
Oldenburg et al. 2001 [26]	CS	43	43	diverse	30.7	nr	nr	nr	27.9	nr	nr	X-ray and CT	ATS 1987	Brändli 1996
Oliver et al. 1988 [56]	CS	383	359	railway	29.2	13.4	35.6	15.0	26.2	23.4	25.1	X-ray	ATS 1979,1987	Crapo 1981
Paris et al. 2004 [17]	CS	706	51	asbestos industry	24.9	9.1	nr	nr	#31.4	nr	nr	X-ray/HRCT	ATS 1986	Quanjer 1993
Petrovic et al. 2004 [18]	CS	120	120	asbestos cement fabric	20.0	9.8	nr	nr	100	-	-	X-ray	CECA 1972	Quanjer 1993
Piirilä et al. 2005 [78]	CS	590	367	diverse	#25.7	9.4	nr	nr	3.0	#21.0	13.7	HRCT	ERS (Quanjer 1992)	Viljanen 1982
Prince et al. 2008 [79]	CS	19	19	diverse	nr	nr	nr	nr	15.8	23.5	14.5	X-ray/CT	ATS 2005	Knudson 1983
Robins and Green 1988 [57]	CS	182	73	asbestos industry	30.2	nr	nr	nr	18.8	22.9	16.3	X-ray	Crapo 1981	Crapo 1981
Rösler and Woitowitz 1990 [19]	CS	144	20	diverse	15.6	6.0	nr	nr	100	-	-	X-ray	according to ERS/ATS	Quanjer 1983
Rui et al. 2004 [61]	FU	103	103	diverse	25.0	7.0	nr	nr	36.0	nr	nr	HRCT	CECA 1971	Quanjer 1983
Schwartz et al. 1990 [58]	CS	1211	1209	sheet metal	32.7	6.7	nr	nr	20.3	26.9	29.4	X-ray	ATS 1972	Knudson 1983
Schwartz et al. 1993 [33]	CS	60	60	sheet metal	>= 1	nr	>= 20	nr	22.0	28.2	23.0	X-ray	ATS 1979	Moris 1971; Goldman 1959
Sette et al. 2004 [80]	CS	87	82	cement/chrysotile miner	#13.4	11.7	nr	nr	nr	#30.7	21.9	CT	ATS 1995	Pereira 1992
Vierikko et al. 2010 [81]	CS	627	86	diverse	#18.2	11.7	#43.3	6,7	#16,9	#15.5	16,9	HRCT	according to ERS/ATS	Viljanen 1982
Zejda 1989 [82]	CS	81	56	asbestos cement industry	17.4	6.9	nr	nr	16.1	nr	nr	X-ray	CECA 1965	Quanjer 1993

Main characteristics of the Studies included in the meta-analysis. SD: standard deviation, CI: confidence interval CC: Case-control, CS: Cross-sectional; FU: follow-up; nr: not reported; m: median; r: range; X-Ray: chest X-ray; HRCT: high resolution computer tomography; CT: computer tomography; #:for the included subjects; §: calculated from SE. *Additional information obtained from [83]

Except for two studies [18,19], all included current and/or former smokers. The proportion of participants reporting to be never-smokers ranged across the studies from only 3% to 100% (median 26.2%), with three studies not reporting the proportion of never-smokers. Smoking severity was reported in 18 of the studies that included smokers and ranged from 14.0 ± 11.9 to 38.9 ± 29.4 pack-years (mean ± SD).

Radiological imaging was done relying exclusively on chest X-ray in 15 studies and relying exclusively on CT or HRCT in 7 studies. Eight studies considered both chest X-ray and CT/HRCT. Mainly VC, FEV_1 _or FEV_1_/VC, or combinations of these parameters, were reported. Some studies provided additional parameters, but due to their scarcity and heterogeneity in assessment methods we did not include them in the meta-analysis. In all studies, lung function test results were acquired according to a quality standard, with the majority (67%) following the American Thoracic Society (ATS) standard procedure available at the time. There was considerable heterogeneity regarding the reference values used to calculate "percent of predicted", with a total of 12 different reference values used across the included studies. The most frequently used reference values were those proposed by Quanjer 1983/1993 [20,21] (n = 5 studies), followed by those of the ATS [22] and Knudson 1983 [23] (both in 4 studies each).

### Quantitative data synthesis

**Figure 2:**
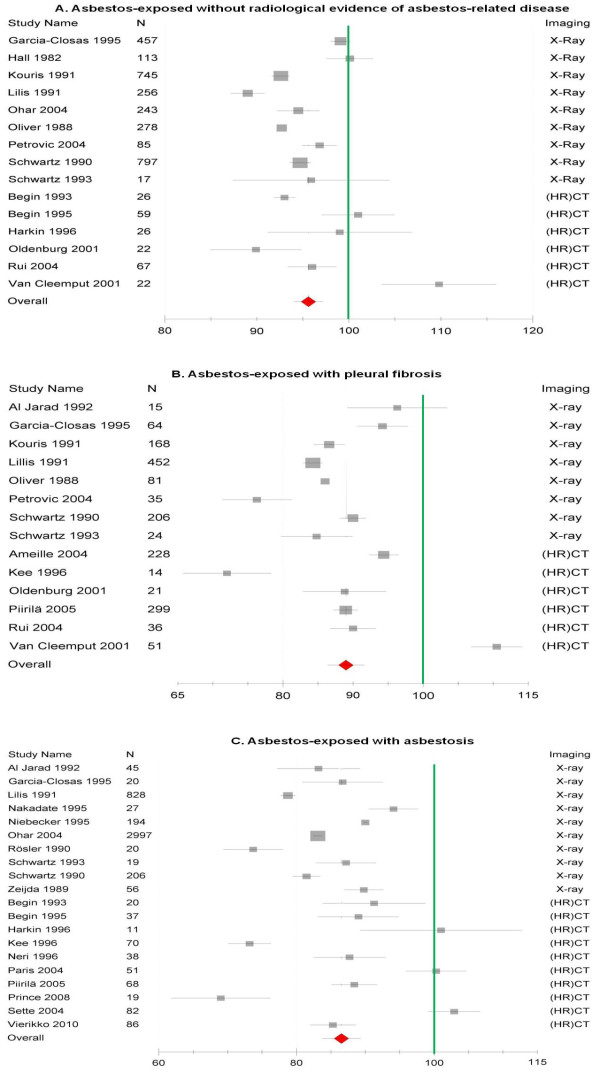
**Forest plot of FVC (expressed as percent predicted with 95%CI) in asbestos-exposed collectives grouped according to the radiological status**. 2A shows the subgroups without asbestos-related diseases, 2B shows the subgroups with pleural fibrosis and 2C shows the subgroups with asbestosis.

**Figure 3:**
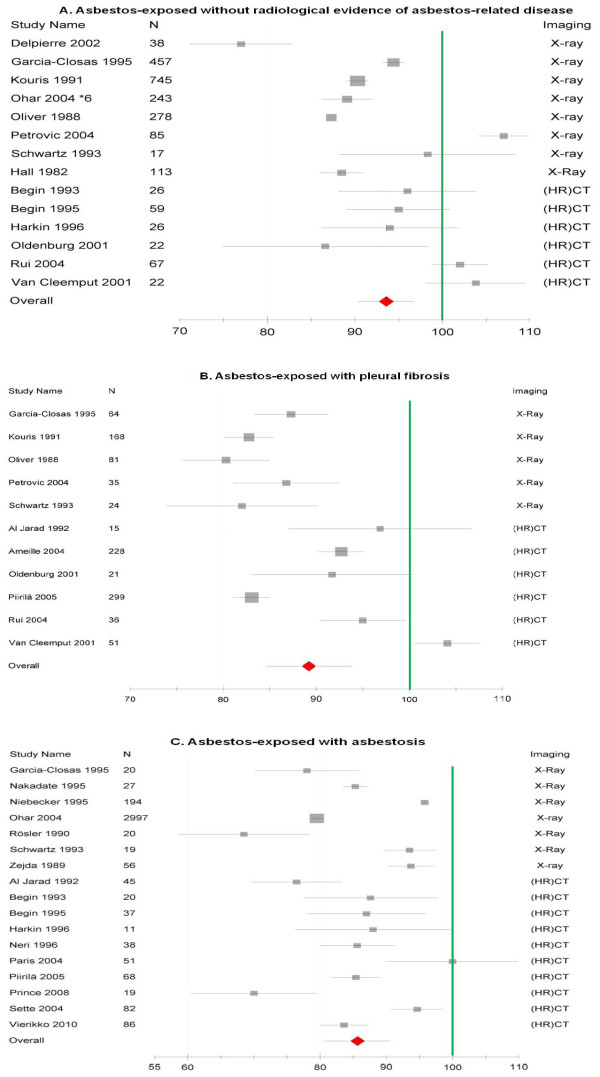
**Forest plot of FEV_1 _(expressed as percent predicted with 95%CI) in asbestos-exposed collectives grouped according to the radiological status**. 3A shows the subgroups without asbestos-related diseases, 3B shows the subgroups with pleural fibrosis and 3C shows the subgroups with asbestosis.

**Figure 4:**
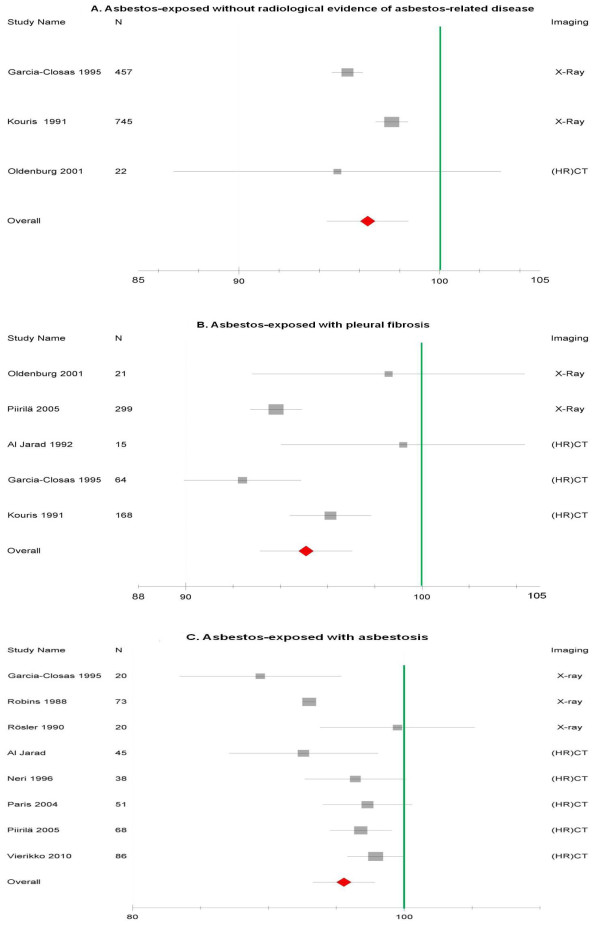
**Forest plot of FEV_1_/FVC (expressed as percent predicted with 95%CI) in asbestos-exposed collectives grouped according to the radiological status**. 4A shows the subgroups without asbestos-related diseases, 4B shows the subgroups with pleural fibrosis and 4C shows the subgroups with asbestosis.

Figures 2, 3 and 4 provide an overview of the pooled estimates of lung function parameters according to radiological findings.

### Vital capacity

Vital capacity (VC, FVC) was the parameter most commonly reported in an adequate manner for inclusion in our meta-analysis. Overall, asbestos-exposed workers showed an impairment of vital capacity when compared with reference values (Figure 2). This impairment of vital capacity was already manifest in workers without radiological evidence of asbestos-related pleural or parenchymal diseases (95.7%-predicted; 95%-CI 93.9, 97.3). The loss of vital capacity was most accentuated in subjects with radiological findings of asbestosis (86.5%-predicted; 95%-CI 83.7, 89.4). The subgroup analysis based on the radiological procedure showed lower estimates of vital capacity in all three radiological categories among studies using conventional chest X-ray compared with those using (HR)CT (Table 2).

**Table 2:** Estimates of lung function according to radiological findings.

	Overall				Studies with X-ray				Studies with (HR)CT			
	
	n	Estimate	95% CI	I^2 ^(%)	n	Estimate	95% CI	I^2 ^(%)	n	Estimate	95% CI	I^2 ^(%)
*FVC (% predicted)*												
Normal imaging	15	95.7	93.9-97.3	94.8	9	94.9	92.9-96.9	96.2	6	97.1	94.2-100.1	89.1
Pleural fibrosis	14	89.0	86.5-91.5	96.1	8	87.1	83.9-90.4	89.5	6	91.6	87.8-95.4	96.8
Asbestosis	20	86.5	83.7-89.4	98.2	10	84.8	80.8-88.8	98.9	10	88.5	84.3-92.7	95.8
*FEV_1 _(% predicted)*												
Normal imaging	14	93.6	90.6-96.5	97.3	8	91.4	87.7-95.1	98.0	6	97.4	92.5-102.2	64.7
Pleural fibrosis	11	89.2	84.7-93.7	93.7	5	83.9	77.2-90.5	42.0	6	93.7	87.6-99.9	95.8
Asbestosis	17	85.7	80.6-90.7	98.8	7	85.5	77.8-93.1	99.5	10	85.8	79.2-92.5	80.8
*FEV_1_/FVC (% predicted)*												
Normal imaging	3	96.4	94.3-98.5	86.9	2	97.4	92.5-102.2	64.7	1	94.9	86.8-103.0	-
Pleural fibrosis	5	95.4	92.7-98.1	68.7	2	93.7	87.6-99.9	95.8	3	96.3	92.6-100.1	68.1
Asbestosis	8	95.5	94.1-96.9	83.8	3	85.8	79.2-92.5	80.8	5	97.0	95.7-98.3	0.0

Comparison of imaging procedure.

Estimates for forced vital capacity (FVC), forced expiratory volume in the first second (FEV1) and the ratio of both parameters (FEV1/FVC) for each radiological subgroup. Results are shown for all included studies as well as separated according to the radiological method used for the diagnosis (conventional chest X-ray or (high resolution) computed tomography. Estimates are expressed as percent predicted together with confidence interval (CI) and I2 as a measure of heterogeneity, n = number of studies included in each subgroup.

Heterogeneity was very high in all three radiological subgroups (I^2 ^>90%) and remained after subgroup analysis according to radiological procedure.

### FEV_1_

As for vital capacity, asbestos-exposed workers showed an impairment of FEV_1 _which was already present in workers with no radiological evidence of asbestos-related disease and was considerably more pronounced in subjects with radiological signs of asbestos-related pleural and/or parenchymal diseases (Figure 3). Again, the subgroup analysis showed differences between studies using chest X-ray and studies using (HR)CT (Table 2). The differences between both imaging procedures were particularly pronounced for subjects identified as having asbestos-related pleural disease. For this group of patients, the estimate of FEV_1 _obtained from the subgroup of studies using conventional X-ray was about 10 percent lower than estimate obtained from HR(CT) studies (83.9%-predicted; 95% CI 77.2, 90.5 vs. 93.7%-predicted; 95% CI 87.6, 99.9) (Table 2).

Heterogeneity was also very high for these analysis (I^2 ^>90%), but decreased to some extent when grouping studies according to radiological technique.

### FEV_1_/VC

FEV_1_/VC was less commonly reported in an adequate manner for inclusion in our analysis. Slight FEV_1_/VC reductions were already seen in workers even without radiological signs of disease, and were similar to those seen for workers with evidence of pleural disease and for those with signs of lung fibrosis related to asbestos (Figure 4). As for the other lung function parameters, there were differences between studies according to the radiological method used, with a tendency to lower FEV_1_/VC among the studies using chest X-ray.

Heterogeneity was considerable (I^2 ^>60%) but not as pronounced as for the other lung function parameters.

### Subgroup analysis and meta-regression

#### Smoking

Few studies reported estimates stratified by smoking status and radiological category. The proportion of never-smokers was reported in 27 studies. The lung function estimates derived from the subgroup analysis showed greater impairment among studies with more than 25% of participants reporting to be never-smokers for subjects without radiological evidence of asbestos-related disease and in those with pleural fibrosis (Table 3). In the group of workers showing radiological evidence of asbestosis lung function impairments were strongest and a bit more pronounced in the subgroup of studies with a lower proportion of never-smokers.

**Table 3:** Estimates of lung function according to radiological findings.

	Overall				Studies with <25% non-smokers				Studies with >25% non-smokers			
	
	n	Estimate	95% CI	I^2 ^(%)	n	Estimate	95% CI	I^2 ^(%)	n	Estimate	95% CI	I^2 ^(%)
*FVC (% predicted)*												
Normal imaging	14	96.1	93.9-98.2	95.1	6	98.1	94.6-101.6	88.0	8	94.9	92.3-97.5	96.6
Pleural fibrosis	12	90.3	87.4-93.3	96.5	6	93.2	88.9-97.5	95.9	6	87.7	83.7-91.8	95.4
Asbestosis	18	86.4	83.2-89.6	98.1	12	85.9	81.9-89.8	83.7	6	87.4	81.9-92.7	98.9
*FEV_1 _(% predicted)*												
Normal imaging	13	93.9	90.0-97.8	97.4	5	97.5	90.9-104.1	35.4	8	92.0	87.2-96.8	98.3
Pleural fibrosis	10	89.9	84.1-95.7	93.6	5	91.5	83.2-99.9	96.3	5	88.5	80.4-96.5	86.2
Asbestosis	16	85.2	81.4-89.1	98.9	11	84.2	79.5-88.8	92.2	5	87.6	80.7-94.4	97.5
*FEV_1_/FVC (% predicted)*												
Normal imaging	2	95.4	94.6-96.2	0.0	2	95.4	94.6-96.2	0.0	-	-	-	
Pleural fibrosis	4	95.4	91.5-99.3	62.5	2	95.9	90.6-101.3	74.9	2	94.9	89.2-110.5	73.2
Asbestosis	8	95.6	93.2-97.9	83.8	4	96.3	94.2-98.4	55.3	4	95.3	92.2-98.3	89.8

Subgroup analysis according to % of never-smokers.

Estimates for forced vital capacity (FVC), forced expiratory volume in the first second (FEV1) and the ratio of both parameters (FEV1/FVC) for each radiological subgroup. Results are shown for all included studies as well as separated according to the proportion of non-smokers included in each subgroup (less ore more than 25%). Estimates are expressed as percent predicted together with confidence interval (CI) and I2 as a measure of heterogeneity, n = number of studies included in each subgroup.

In the regression analysis of the effect of the proportion of non-smokers on estimates of FEV_1_, those studies with a higher proportion of never-smokers tended to show less impairment of this parameter (not statistically significant) for all three radiological categories.

Table 4 shows the results of three studies [24-26] reporting estimates for non-smokers and smokers without radiological evidence of parenchymal disease. These papers suggest mainly a synergistic effect of smoking and asbestos exposure.

**Table 4:** Asbestos-exposed workers without radiological evidence of parenchymal disease stratified by smoking status.

		Non-smokers			Smokers		
Studie		n	% predicted	SD	n	% predicted	SD
Hall 1982	FEV_1_	46	101.0	13.6	67	92.5	14.9
	FVC		102.2	11.6		99.2	13.4
Neri 1996	FEV_1_	34	90.9	15.6	47	92.0	14.0
	FVC		89.7	14.9		90.9	14.3
	FEV_1_/FVC		100.3	10.9		100.2	6.8
Oldenburg 2001	FEV_1_	12	105.7	13.6	31	83.6	25.1
	FVC		96.1	10.9		86.7	12.6
	FEV_1_/FVC		102.3	4.39		94.5	18.6

Differences in forced vital capacity (FVC), forced expiratory volume in the first second (FEV1) and the ratio of both parameters (FEV1/FVC) between asbestos exposed non-smokers and smokers without radiological evidence of asbestosis. Estimates expressed as percent predicted together with standard deviation (SD) and I2 as a measure of heterogeneity, n = number of subjects included in each subgroup.

#### Duration of asbestos exposure

Mean exposure duration was reported in 23 studies. The data was heterogeneous (Table 5). FEV_1 _was consistently better across all radiological categories in the subgroup of studies with a mean exposure length of more than 22 years. In contrast, FEV_1_/VC was consistently better across all radiological subgroups for the studies with shorter mean exposure duration. The results for FVC were inconsistent. The regression analysis, however, indicated that lower FVC and FEV_1 _could be expected with increasing mean exposure duration.

**Table 5:** Estimates of lung function according to radiological findings.

	Overall				Studies <22 yr. mean exposure				Studies >22 yr. mean exposure			
	
	n	Estimate	95% CI	I^2 ^(%)	n	Estimate	95% CI	I^2 ^(%)	n	Estimate	95% CI	I^2 ^(%)
FVC (% predicted)												
Normal imaging	11	96.2	94.4-98.0	95.9	4	97.0	94.2-99.8	96.5	7	95.7	93.4-98.0	90.8
Pleural fibrosis	11	89.2	85.6-92.8	96.9	2	81.8	73.2-90.3	92.8	9	90.8	86.8-94.8	98.0
Asbestosis	12	87.4	82.2-92.6	95.5	5	87.9	79.9-95.9	96.1	7	87.0	80.2-93.9	95.0
FEV_1 _(% predicted)												
Normal imaging	11	93.7	89.3-98.1	97.9	5	91.8	85.5-98.1	97.4	6	95.5	89.3-101.7	96.1
Pleural fibrosis	9	89.2	83.9-94.5	94.8	2	84.7	73.5-95.8	35.5	7	90.6	84.6-96.5	95.5
Asbestosis	10	86.8	82.3-91.2	84.2	5	86.4	80.3-92.5	90.4	5	87.1	80.6-93.6	66.7
FEV_1_/FVC (% predicted)												
Normal imaging	3	96.4	94.3-98.5	86.9	2	96.5	94.3-98.7	93.4	1	94.9	86.2-103.6	-
Pleural fibrosis	4	95.5	92.9-96.2	68.2	1	96.2	94.4-97.8	-	3	93.8	91.9-95.8	48.1
Asbestosis	7	95.8	93.8-97.9	86.1	3	97.7	95.9-99.5	0.0	4	94.6	92.0-97.2	83.2

Subgroup analysis by mean exposure duration.

Differences in forced vital capacity (FVC), forced expiratory volume in the first second (FEV1) and the ratio of both parameters (FEV1/FVC) between subgroups with a mean exposure duration of less (<22 yr.) and more for than 22 years (>22 yr.). Results are shown for each radiological subgroup. Estimates are expressed as percent predicted together with confidence interval (CI) and I2 as a measure of heterogeneity, n = number of studies included in each subgroup.

## Discussion

Several population-based studies provide evidence of asbestos exposure contributing significantly to the burden of airway diseases, but a detailed assessment of exposure was generally neither presented nor performed in such studies [27-29]. The pleural plaque incidence in the general population is in the range of 0.02 to 12.8% [30] and is 80-90% attributable to asbestos exposure [31]. The initial concern about the potential adverse effects of asbestos on lung function was vindicated in clinical as well as epidemiologic studies over many years [12,13]. The present meta-analysis has considered the major lung function parameters VC, FEV_1_, FEV_1_/VC, for asbestos-exposed workers grouped, according to their radiological diagnosis, into three groups: "absence of pleural and lung parenchymal fibrosis", diagnosed with "pleural fibrosis" (PP and/or DPT) or "asbestosis with or without pleural fibrosis". Overall, our analysis shows a statistically significant reduction of VC, FEV_1 _and FEV_1_/VC among workers exposed to asbestos compared to the general population (i.e. reference values).

The severity of the observed impairments is related to the degree of radiological abnormalities indicative of pleural fibrosis and asbestosis. Overall, VC and FEV_1 _scores were lowest for those workers showing radiological findings of asbestosis, followed by those with signs of pleural fibrosis. Workers exposed to asbestos with normal radiological findings (either X-ray or (HR)CT) exhibited significantly better VC and FEV_1 _scores than those with radiological abnormalities, but their decreased values indicate some degree of lung function impairment. FEV_1_/VC was slightly reduced in all groups. This reduction was more evident in the subgroups with radiological abnormalities. These differences between groups persisted mostly when the studies were analysed separately, according to the radiological methods used (either X-ray or (HR)CT), although less pronounced for the (HR)CT-based studies of the three subgroups of patients. In general, studies with (HR)CT based diagnosis report milder lung function impairments than those using conventional X-ray due to the higher sensitivity of the (HR)CT for mild grades of pleural disorders and asbestosis.

A positive relationship between the severity of functional impairment and the radiologically defined degree (score) of asbestos-related pleural and/or pulmonary fibrosis was already reported in a few studies [32-34]. As shown the absence of characteristic radiological findings does not exclude lung function abnormalities. Our meta-analysis revealed statistically significant deterioration in the lung function parameters for asbestos workers without any evidence of radiological abnormalities. These findings extend the meta-analysis by Filippelli, Martines et al [35] who found statistically significant reductions in all investigated lung function parameters in subjects exposed to asbestos, although the authors did not account for different radiological findings. Regression models reported in some of the included studies indicate that the radiological findings can only explain a small part of the variability in these parameters. Other authors have also reported a medium to low explanatory power of radiological findings for other lung function parameters [33,32].

There is evidence from clinical studies that discrepancies between lung function and radiological findings can be due to asbestos-induced pulmonary alterations not radiologically detectable. These studies describe multiple cellular lesions, apoptosis, inflammatory and profibrogenic responses, using histopathology and electron microscopy, as well as the synthesis of associated mediators and oxygen radicals [36-40]. It has been estimated that exposure to an asbestos fibre dose [41] of 25 fibre-years represents the inhalation of about 55 billion asbestos fibres [42], of which a significant proportion is deposited in the lung.

Our findings indicate not only the presence of restrictive but also of obstructive ventilation patterns in workers exposed to asbestos, either with or without asbestos-related radiological abnormalities: an issue of controversial discussion.

Recently, Dement et al. [43] found an overall COPD prevalence of 18.9% in asbestos workers/insulators. In their collective of older construction and trade workers, at the US Department of Energy with mixed exposure at nuclear sites, the prevalence of COPD was of 23% among those only with pleural changes and 32.3% among those with both pleural and parenchymal changes [43]. Conversely, Ameille et al. [44] reported a lack of association between occupational exposure to asbestos and airway obstruction. They determined that FEV_1_/FVC and FEV_25-75 _did not differ through the cumulative exposure classes and there was no significant correlation between cumulative exposure to asbestos and pulmonary function parameters nor with the proportion of abnormal pulmonary function tests [44]. However, these authors did not include a non-exposed control group and report generally elevated values for FVC, FEV_1_, FEV_1_/FVC and residual volume (RV), which can be explained by the selected study population (volunteers for a screening programme without previous severe respiratory disease).

### Bias and limitations

The degree of lung function impairment may have been underestimated due to bias in the included studies. Two main sources of not negligible underestimation of adverse health effects in actual occupational cohort studies are the dilution effect and the comparison bias [45]. The dilution effect results from the inclusion of not or very low exposed workers in the study cohort. The comparison bias results from a healthy hire effects at the beginning of exposure history. The lung function of blue collar workers - like the ones included in our study - is typically better than the references taken from the general population (i.e. over 100% predicted) [46,47]. In those workers lung function values studied at a single time point may be still within the norm despite an underlying considerable absolute decrease since the start of exposure (e.g. a FEV_1 _fall from 115% to 95%). Comparison bias results also from the healthy worker effect in the course of the working life. Subjects with relevant health impairments may change their occupation or have a shortened work life and thus may not be available for recruiting to later lung function assessment based on occupation or worksite. For example Fell et al. [48] hypothesized in their investigation on respiratory symptoms and ventilatory function of workers exposed to cement dust that individuals susceptible to adverse respiratory effects from cement dust may have quitted work and therefore dropped out of the exposed groups. The authors found a high prevalence (55%) of respiratory symptoms and COPD in the group of former cement workers visited at home, underlying the importance of included former workers. These biases are probably present in the studies included in our systematic review, since most of them had a cross-sectional design not accounting for changes in lung function over time and in general did not consider former workers.

In our meta-analysis, there is a high degree of heterogeneity (high I^2^) across the studies, which we acknowledged by using a random effects model. Heterogeneity is caused by variations in the individual study populations as well as differences in study methods.

With respect to the study design, a major source of heterogeneity is the quality of lung function tests and the variety of references values used in the studies. We included predicted values, as given by the various authors with their considerable variation. For example, the reference values of Quanjer et al. [20,21] have been shown to be at least 10% too low for current normal populations [49-53], thus leading to an underestimation of the effects of asbestos exposure. The same is true for some other reference values based on inadequate reference populations.

The issue of the study population as a source of heterogeneity includes the following aspects: First, studies differed considerably in the duration of occupational exposure to asbestos, ranging from less than 1 year to over 30 years. The subgroup analysis indicated that the results for FEV_1 _and for FEV_1_/VC were negatively related to the duration of exposure. The meta-regression analysis indicated an inverse relationship between exposure duration and FVC and FEV_1 _(i.e. lower estimates with increasing mean exposure duration). However, this can only explain a small amount of heterogeneity. There are also major differences between studies regarding the intensity of exposure because of the wide variety of tasks and occupations studied. Since only two studies [41,54] reported an estimation of exposure intensity (i.e. fibre-years), we could not explore this source of heterogeneity in subgroup or regression analysis. Similarly, mean latency times were only reported in nine of the included studies, thus subgroup analysis or meta-regression to explore heterogeneity could not be performed.

An additional source of heterogeneity may be the differences in the distribution of confounders, such as smoking or co-exposure to other occupational noxae. Regarding co-exposures most of the studies provided little information and we could not explore this potential source of heterogeneity in detail.

An important question concerns the interaction between smoking and asbestos exposure. Only a few studies accounted for smoking in their analysis appropriately. In one of the two studies that included only never-smokers [18], reduced VC was reported for both asbestos-exposed workers without and with pleural fibrosis, and an impairment of FEV_1 _was seen in those with pleural fibrosis. The other study considering only never-smokers examined patients with asbestosis. Here all lung function parameters were correspondingly impaired [19].

Niebecker and colleagues showed for patients with asbestosis that the degree of impairment was greater among smokers [9]. Some of the included studies [16,33,55-61] reported multivariate linear regression models including smoking as an explanatory variable (among others). The results of these analyses suggest an association of lung function impairments with pleural abnormalities independent of smoking, i.e. when pleural fibrosis is present then impairments in lung function can be observed in both smokers and non-smokers.

At the study level, the results of subgroup analysis according to the proportion of never-smokers were inconsistent and partly counterintuitive, since for some parameters, the higher the proportion of non-smokers in a study, the lower were the estimates. An additional analysis using the mean pack-years - as an indication of the dose - was not performed, because one third of the included studies did not report the information.

Therefore our approach does not allow a clear differentiation of smoking effects from those of asbestos, mainly due to the shortcomings or the failure to report findings of the included studies but provides evidence that the observed impairment in lung function in the absence of radiological signs of asbestos-related parenchymal disease cannot be attributed solely to smoking and that asbestos exposure plays a causal role.

A recent meta-analysis [35], which did not consider radiological findings, demonstrated independent significant effects of smoking as well as of asbestos exposure (i.e. a synergistic effect), both for forced expiratory flow (FEF_25-75_, FEF_50_) as well as thoracic gas volume (TGV) and RV/TGV. In this analysis, the influence of asbestos exposure was stronger than that of smoking for FEV_1_/VC and airway resistance, whereas smoking had a stronger effect on FEF_25-75_. Evidence for a synergistic detrimental effect of smoking and asbestos exposure on airflow limitation has also been reported in several additional studies (FEV_1 _[62,41,61,64], FEV_1_/VC [65,66,9,10,4,25,61,26], FEF_25-75 _[66,3,25,10,43] and FEF_75-85 _[66,3]).

It has to be acknowledged that our study does not allow answering the question whether the observed statistically significant lung function impairments at the population level are also of clinical relevance at the individual level. Indeed, in clinical practice the diagnosis of an obstructive defect requires a FEV_1_/FVC of less than 70% and a FEV_1 _over 80% from predicted is considered to represent mild impairment in an individual [67]. Our pooled estimates are within the normal limits applied to individuals (even when considering the lower limits of the confidence interval). Small decreases in group mean values however do not preclude clinically important disease. For example a group of workers exposed to asbestos with moderate dyspnoea had mean FVC of 96%, mean FEV_1 _of 94% and mean FEV_1_/FVC of 95% of predicted [68], which are similar to our pooled estimates. In one study, lung function impairments, particularly airflow obstruction, have been associated with increased mortality in asbestos exposed workers [69].

## Conclusions

We conclude that asbestos exposure causes restrictive as well as obstructive lung function impairment. Asbestos-exposed workers may present lung function impairments even in the absence of radiological evidence of asbestos-related pleural fibrosis or asbestosis.

Our systematic review demonstrates that despite the large number of studies about the health hazards from occupational exposure to asbestos, there is a need for further research, especially on the role of smoking, occupational co-exposure (e.g. other mineral dusts, welding fumes) and possible synergistic effects on the development of functional impairment, particularly chronic airway obstruction, in asbestos-exposed workers. Such studies should include measurement of CO diffusion capacity, airway resistance and flow volume curves in a consistent approach. Furthermore, our study underlines the necessity for an international agreement on lung function reference values within the individual ethnic groups, to facilitate comparison between different studies.

## Abbreviations

CI: confidence interval; D_L, CO_: CO diffusion capacity; DPT: diffuse pleural thickening; FEF: forced expiratory flow; FEV1: forced expiratory volume in the first second; FVC: forced vital capacity; HRCT: high resolution computed tomography; PP: pleural plaques; RV: residual volume; SD: standard deviation; SE: standard error; SVC: slow (relaxed) vital capacity; TGV: thoracic gas volume; TLC: total lung capacity; VC: vital capacity; X-ray: chest radiograph

## Competing interests

The authors declare that they have no competing interests.

## Authors' contributions

All authors had full access to all data. XB had the original idea for the paper and vouches for the integrity of the analysis. DW, UM and MVG extracted and analysed the data. All authors collaborated in interpreting the data and writing the manuscript and read and approved the final manuscript.
